# [^*18*^F]FDG Positron Emission Tomography for Initial Staging and Healing Assessment at the End of Therapy in Lymph Nodes and Bone Tuberculosis

**DOI:** 10.3389/fmed.2021.715115

**Published:** 2021-08-17

**Authors:** Laure Sarda-Mantel, Jidar Kaoutar, Toni Alfaiate, Amanda Lopes, Frédéric Paycha, Khadija Benali, Nidaa Mikail, Michael Soussan, Charles Lemarignier, Frédéric Méchaï, Sophie Le Nagat, Françoise Montravers, Ouda Deradji, Emmanuel Durand, Tiphaine Goulenok, Diane Ponscarme, Patrick Yéni, Cédric Laouénan, Christophe Rioux

**Affiliations:** ^1^Nuclear Medicine Department, Lariboisière Hospital, APHP, Paris, France; ^2^Infectious Diseases Department, Bichat Hospital, APHP, Paris, France; ^3^Université de Paris, INSERM, IAME UMR 1137, Paris, France; ^4^Internal Medicine Department, Lariboisière Hospital, APHP, Paris, France; ^5^Nuclear Medicine Department, Bichat Hospital, APHP, Paris, France; ^6^Nuclear Medicine Department, Avicenne Hospital, APHP, Bobigny, France; ^7^Nuclear Medicine Department, Saint-Louis Hospital, APHP, Paris, France; ^8^Infectious Diseases Department, Avicenne Hospital, APHP, Bobigny, France; ^9^Infectious Diseases Department, Tenon Hospital, APHP, Paris, France; ^10^Nuclear Medicine Department, Tenon Hospital, APHP, Paris, France; ^11^Internal Medicine Department, Bicêtre Hospital, APHP, Le Kremlin Bicêtre, France; ^12^Nuclear Medicine Department, Bicêtre Hospital, APHP, Le Kremlin Bicêtre, France; ^13^Internal Medicine Department, Bichat Hospital, APHP, Paris, France; ^14^Infectious Diseases Department, Saint-Louis Hospital, APHP, Paris, France; ^15^Université de Paris, INSERM, IAME UMR 1137, Paris, France

**Keywords:** tuberculosis, bone, lymph nodes, [^18^F]FDG-PET, Positron emission tomography, antibiotherapy monitoring

## Abstract

**Objective:** In extra-pulmonary tuberculosis, therapeutic management is difficult in the absence of reliable tool to affirm healing at the end of treatment. In this prospective multicenter study, we evaluated [^18^F]FDG-PET for this purpose.

**Methods:** Forty-two patients out of 55 included patients could be analyzed. Additionally to usual biological, histological and morphological explorations, [^18^F]FDG-PET was performed at diagnosis (PET1), at the end of treatment (PET2), indeed 6 months later. Then patients were followed until 12 months after end of prescribed treatment.

**Results:** PET1 was positive in 97.6% of patients and discovered unknown injured sites in 52.7% of cases. PET2 was positive in 83.3% of uncured patients, and in 82.3% of cured patients. The sum and mean value of SUV_max_ measured in PET/CT lesions decreased between PET1 and PET2 in all patients. Mean value of SUV_max_ (MSUV) and sum value of SUVmax on PET2 showed the highest AUC on ROC curves for the diagnosis of healing at the end of prescribed treatment; MSUV 3.5 on PET2 had a sensitivity of 76.5% and a specificity of 80.0% to affirm healing at the end of prescribed treatment.

**Conclusions:** [^18^F]FDG-PET/CT was useful at diagnosis, discovering unknown lesions in 52.7% of cases. MSUV on PET2 was the best criteria to affirm healing at the end of prescribed treatment.

## Introduction

Tuberculosis remains a major public health problem worldwide with more than 9 million cases per year in 2009 (137/100,000 inhabitants). Its endemic evolution associated with the explosion of HIV in emerging countries, particularly in sub-Saharan Africa, has increased the number of new annual cases of the disease since 1990 from 6.6 to 9.4 million (1). Mortality remains worrying with 1.7 million deaths worldwide, more than half of them in Africa. The first region affected in metropolitan France is the Ile de France with 36% of reported cases, an incidence of 17.9/100,000 inhabitants. The two most affected departments are Paris and the department of Seine-Saint-Denis (respectively, 27.5 and 30.3/100,000 inhabitants). At-risk groups with higher incidence are identified: people from sub-Saharan Africa (159.1/100,000 inhabitants), homeless people (223.1/100,000 inhabitants), elderly people (16.9/100,000 inhabitants for the over 75 years). The data of the declaration in France concerning the anatomical localization of the tuberculosis are rather restricted. Also in 2008, it was pulmonary tuberculosis associated or not with another localization in 70.4% of the cases; of the remaining 27.8, 51.4% (*n* = 824) were pleural or intra-thoracic lymph nodes, 5.7% were tuberculous meningitis, and 7.7% were tuberculous miliaries (2).

The definitive diagnosis of tuberculosis can only be made on the identification of the mycobacterium in culture with the presence of one of the three species belonging to the tuberculosis complex: *Mycobacterium tuberculosis, M. africanum*, and *M. bovis*. In pulmonary tuberculosis, the diagnosis remains relatively easy. In extra-pulmonary forms, on the other hand, cultures are much more often negative. The few available French data show that in extra-pulmonary tuberculosis sites for which a puncture could be performed, the cultures are positive in, respectively, 38, 70, 25, and 42% of cases of lymph node, bone, pleural and meningeal injury; whereas in more than 90% of cases they are pulmonary forms (3).

In pulmonary tuberculosis, the negativation of BK tubages after a 3 months treatment is a good indicator of healing. While in pulmonary tuberculosis treatment and follow-up are well-codified (WHO 1997, UICT 2000, ATS 2002) (4), they are less clear in extra-pulmonary forms. Moreover, it is often impossible to confirm by bacteriology the sterilization of the initial sampling site when it has been informative. The management of radiological abnormalities [Computed Tomography (CT), Magnetic Resonance Imaging (MRI)] is not rigorously codified and their persistence at the end of treatment is not systematically synonymous with failure. Indeed, the evolution of imaging is often delayed compared to that of the clinic and radiological healing criteria are poorly defined. Thus, the recommendations in terms of duration of treatment of extra-pulmonary forms remain unclear: at least 6 months for lymph node tuberculosis, between 6 and 9 months for bone/articular tuberculosis, between 9 and 12 months for a neuro-meningeal injury (5). The total duration of treatment is left to the appreciation of the clinician, who in the absence of certainty tends to prolong treatment rather than shorten it. The consequences in terms of individual health (duration of treatment, side effects) and public health (mobilization in human and financial health resources) posed by the uncertainties which concern the positive diagnosis or the diagnosis of cure, as well as the duration of treatment, raise the need for other assessment tools in the management of extra-pulmonary tuberculosis.

During those last years, recommendations and uses of [^18^F]Fluoro-desoxy-glucose Positron Emission Tomography coupled with CT ([^18^F]FDG-PET/CT) have extended from oncological indications to imaging inflammatory diseases. Indeed activated inflammatory cells in infection foci as well as live bacterias have increased glucose metabolism and show increased [^18^F]FDG uptake on [^18^F]FDG-PET scans. [^18^F]FDG accumulation in active tuberculosis foci has been widely reported, as well as its decrease under antibiotic therapy (6, 7). [^18^F]FDG-PET/CT has an excellent predictive negative value for non-active lesions. But in extra-pulmonary tuberculosis, the few studies available report cases of residual [^18^F]FDG uptake in cured patients at the end of antibiotherapy. So the question whether [^18^F]FDG-PET/CT is a reliable tool or not for healing assessment is still unsolved (8, 9).

The aim of this study was to evaluate [^18^F]FDG-PET/CT before and after anti-tuberculosis treatment, assuming that this technique could provide useful data for therapeutic monitoring.

We described [^18^F]FDG-PET/CT evolution between initial diagnosis, end of antibiotherapy indeed 6 months after the end of therapy, and identified PET criteria to affirm or invalidate healing at the end of the therapeutic course.

## Methods

This is a French multicenter prospective pilot study conducted in the seven investigative centers, within the departments of infectious and tropical diseases, internal medicine, and rheumatology, registered in clinicaltrials.gov NCT01613196. The study has been approved by the French ethics committee CPP Ile-de-France 1 (and sponsored by Assistance Publique Hôpitaux de Paris) and the subjects gave informed consent to the work.

### Patients

The inclusion criteria were: Male or Female over 18, Patient who has not been infected with HIV or has been infected with HIV with a CD4 count > 200/mm3 for at least 3 months, Patient with certain or probable lymph node or bone tuberculosis (certain: presence of bacillus acid-alcohol-resistant in collected samples (ganglionic puncture, bone biopsy puncture, but also other samples—in particular pulmonary—in case of associated extra-lymph nodes or bone localizations; probable: cluster of suggestive arguments among which epidemiological context and/or general clinical signs and/or extra respiratory and/or compatible biological and/or radiological abnormalities and start of antituberculous treatment and absence of argument for another etiology possible).

The patients whose tuberculosis was not confirmed on the evolution were secondary excluded.

Exclusion criteria were: Relapse of tuberculosis (patient having already been treated in the past), Suspicion of another concomitant systemic infection (bacterial, fungal or parasitic), Severe immunodepression, Active or progressive neoplasia (solid cancer and hematology), Extended corticotherapy (corticosteroid therapy> 20 mg/day) for at least 3 months, Chronic inflammatory diseases, Pregnant or lactating woman or during periods of genital activity without contraception.

### Treatment

The choice of treatment was made in accordance with the recommendations of the Superior Council of Public Hygiene and the High Authority of Health concerning the management of tuberculosis (10). It consisted in an association of 3 or 4 anti-tuberculosis agents for the first 2 months, followed by a dual therapy for 4–7 months for lymph node locations and 10 months for bone sites. Those are the expected theoretical durations of treatment. In the case of resistance to anti-tuberculosis drug, the treatment was adapted according to the antibiogram data, according to the recommendations (5, 11) and according to the opinion of the reference center of resistance to anti-tuberculosis drugs in case of multi-resistance. The duration and the decision to stop treatment was left to the discretion of the clinician.

### [^18^F]FDG PET Imaging

[^18^F]FDG-PET/CT scans were performed on clinical PET/CT devices in five nuclear medicine departments of Assistance-Publique-Hopitaux de Paris. For each patient, initial, post-treatment, and delayed PET-scan procedures were identical (injected dose, PET/CT device).

Included patients underwent 2 or 3 successive [^18^F]FDG-PET/CT examinations: PET1 within 30 days after initial diagnosis and 15 days after antibiotherapy's initiation, PET2 within 15 days after the end of prescribed treatment, PET3 6 months after the end of treatment if PET2 was positive for tuberculosis.

#### Acquisitions

Patients were asked to fast at least during 8 h before the PET/CT scan. Upon their arrival in nuclear medicine departments, Capillary glycemia was measured before allowing (or not) the PET scan: a glycemia ≤ 9 nmol/ml was mandatory. [^18^F]FDG was injected intravenously at the dose of 3–5 MBq/Kg. Then the patients were asked to keep lying and calm during 1 h until the acquisition. The recording of the images was started 60 min after the injection and included CT acquisition followed by the PET recording. CT recording, necessary for the attenuation correction of PET images as well as the anatomical identification of lesions detected by PET, was performed without contrast injection with voltages around 100 kV and intensities of ~140 mA, in order to obtain axial sections of adequate quality with a thickness ranging from 3 to 5 mm and a matrix 512 × 512. Static PET acquisitions were performed in 3D mode and with a spatial resolution of <5 mm. They were started at the level of the root of the thighs and included several consecutive recordings to cover the pelvis, the abdomen, the thorax and the head. The PET images were reconstructed using an iterative method (OSEM), with parameters allowing to obtain a voxel size ≤ 4 mm in the 3 dimensions of the space. PET images were analyzed using specific softwares, allowing the display of merged images.

#### [^18^F]FDG-PET/CT Analysis

The [^18^F]FDG foci were visually detected according to the criterion of a maximum activity clearly greater than that of the surrounding tissue activity. Only hypermetabolic abnormalities present on both uncorrected and attenuation corrected images were considered significant. Quantitative analysis of hypermetabolic abnormalities was performed using manually drawn regions of interest. The maximum activity in the regions of interest was determined [Maximal Standard Uptake Value (SUV _max_) in g/ml]. The results of [^18^F]FDG-PET/CT at each time were transmitted to the clinician only after he had made his own diagnosis and had completed the results of his diagnosis in the observation book. At the end of the study, centralized reading of all anonymous PET/CT scans was done by four senior nuclear medicine physicians unaware of any other data. The results were recorded in the scorecard including both the number of detected lesions and the highest SUV_max_ measured in each following areas: cervical, axillary, mediastinal and abdominal lymph nodes areas, axial bone, peripheral bone, lungs, abdominal organs, brain, muscles, skin, and subcutaneous soft tissues. A consensus was made in case of discrepancy between the lecturers.

For each PET scanner, SUV_max_ was recorded for each anatomical regions where abnormal hot spot(s) was (were) seen. Then two quantitative criteria were determined on all PET scans: the sum of all recorded regional SUV_max_ (∑SUV), the mean value of recorded regional SUV_max_ (MSUV). Additionally, a lesion by lesion analysis was performed in the patients for whom the information of healing or uncured disease at the end of treatment could be obtained. For such analysis, the highest SUV_max_ value measured on PET2 was considered.

### Healing or Residual Disease Assessment at the End of Prescribed Therapy

Patients were followed up to 12 months after the end of treatment, i.e., 18–24 months after inclusion (duration of treatment: 6, 9, or 12 months depending on the type of injury). Each consultation (Cs1 at initial diagnosis, Cs2 at the end of treatment, Cs3 6 months then Cs4 12 months after the end of treatment) included an examination of the general condition and the general and local signs of infection, weight evaluation, and targeted assessments based on initial locations of tuberculosis. Patients benefited from the usual biological, histological, and morphological explorations at the time of diagnosis and during therapeutic follow-up.

Patients were considered cured if they have been treated for at least 80% of the prescribed time with BK-sensitive drugs, presented no biological or clinical sign of tuberculosis at the end of treatment and have not relapsed 1 year after the end of treatment.

### Statistical Analysis

#### Descriptive Analysis

Patient's characteristics were described using frequency and percentage for categorical variables, and mean and standard deviation or median and inter-quartile range values for continuous variables, depending of the normality of their distribution.

#### Primary Analysis

∑SUM and MSUV were compared between PET1 and PET2 using Wilcoxon signed rank tests.

#### Secondary Analyses

Thepercentages of variation of ∑SUM ([∑SUM on PET2 – ∑SUV on PET1]/∑SUV on PET1) and MSUV were compared between cured and uncured patients using Wilcoxon tests. Pre-specified subgroup analyses were performed in patients with lymph node lesions, and bone lesions. Comparisons also involved Wilcoxon tests.

The evolutions of ∑SUM and MSUV for each patient were described with spaghetti plots. Thus, we analyzed these using a linear mixed effect model. These models had two parameters, one for the baseline sum or mean of the SUV_max_, and one for the slope of evolution of sum or mean of SUV_max_, both with random effects. Parameters were estimated using the REstricted Maximum Likehood (REML) algorithm implemented in SAS 9.4. For each parameter we reported the estimated mean and standard deviation (SD) of inter-individual variability. The predictions of these models were represented in the spaghetti plots. A first analysis was performed among cured patients and followed by a second analysis among uncured patients.

The ROC test curve analysis and the Youden Index were performed for determining the optimal cut-off value for ∑SUM and MSUV at the end of treatment (PET2) to diagnose the recovery.

A *p*-value of < 0.05 was considered statistically significant.

All the analyses were performed using SAS V.9.4 (SAS Institute Inc., Cary, North Carolina, USA).

## Results

### Patients

#### Flow-Chart of the Study

**Figure 1:**
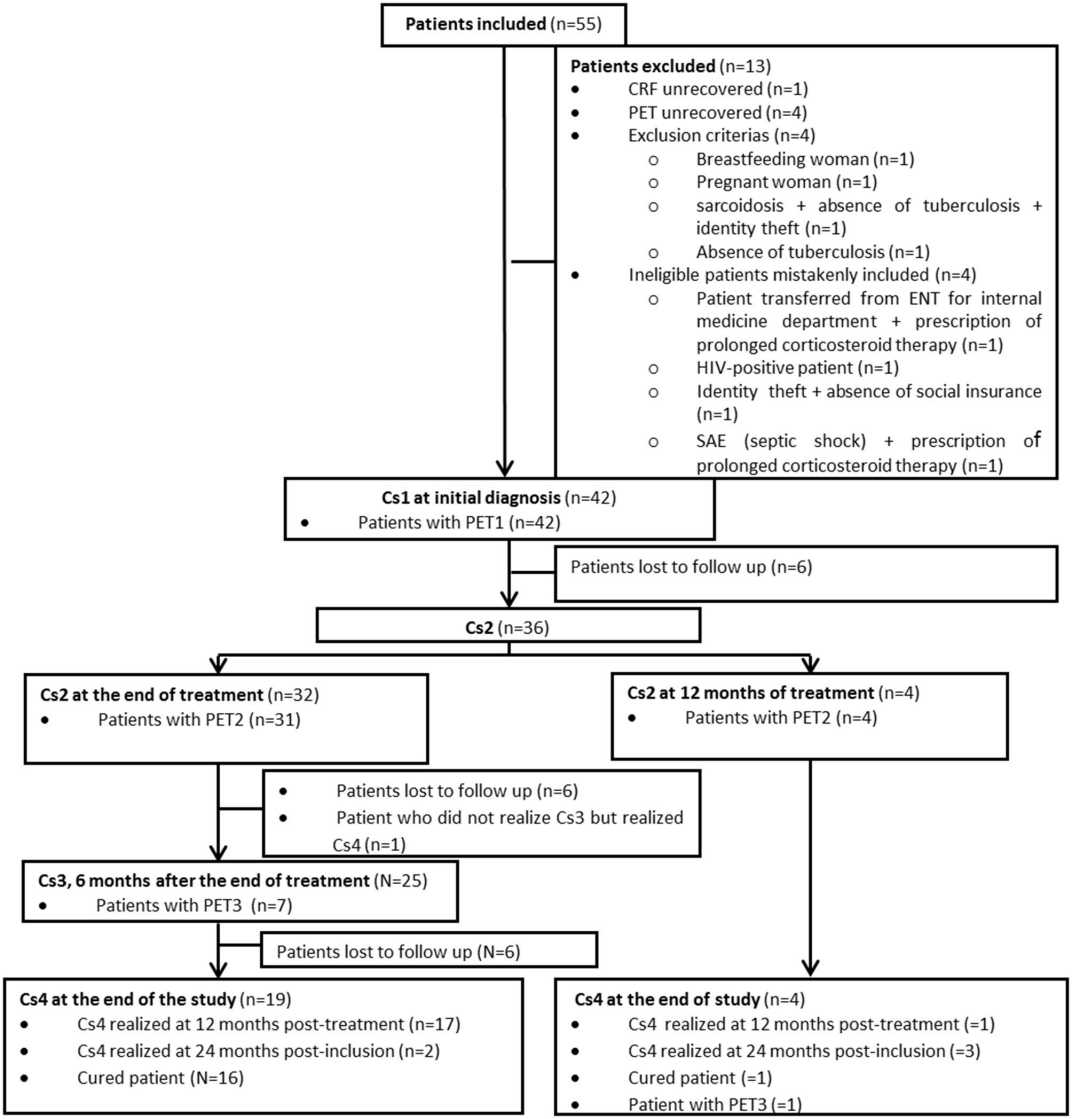
Flowchart of the study. Cs, medical consultation; PET1, Pet performed at initial diagnosis; PET2, PET performed at the end of treatment; PET3, PET performed 6 months after the end of treatment.

The flow-chart of the study is presented in Figure 1. Globally, 55 patients were enrolled in the study from May 2012 to August 2014. Thirteen of them were secondarily excluded (HIV confirmed in one case, tuberculosis not confirmed in two cases, prolonged corticotherapy >20 mg/day established in two cases, CRF unrecovered in one case, PET unrecovered in four cases, absence of social insurance in one case, one breastfeeding woman, and one pregnant woman) so 42 of the 55 included patients could be analyzed. Also, the assessment of healing or residual disease at the end of therapy was not possible in 19 patients either because they were lost to follow-up (no Cs4) or because they did not undergo TEP2 or because they did not undergo Cs2. Also only eight patients with positive PET2 underwent PET3.

The characteristics of analyzed patients are presented in Table 1.

**Table 1:** Description of the study population.

**Inclusion**	***N* = 42**
**Sex**	F: 14 (33.3%), M: 28 (66.7%)
**Age**	39.0 [31.0–49.0]
**Type of tuberculosis**	Lymph nodes: 17 (40.5%)
	Bone: 5 (11.9%)
	Lymph nodes + Bone: 6 (14.3%)
	Lymph nodes + Lung: 6 (14.3%)
	Bone + Lung: 4 (9.5%)
	Lymph nodes + Bone + Lung: 4 (9.5%)
**Associated disease**	Diabetes Type II: 3 (7.1%)
	Chronic inflammatory disease (controlled): 2 (4.8%)
	Cancer: 1 (2.4%)
**Temperature**	37.0 [36.8–37.2]
**Body weight, kg**	63.0 [55.0–80.0]
**Symptoms**	Sweats: 13 (31.0%): Cough: 7 (16.7%); Pain related to tuberculosis: 23 (54.8%)
**Biology**	Hb: 12.1 [11.1–13.7]; WBC: 7.1 [5.6–9.0]; Platelets: 309.0 [265.0–371.0]
	ALAT: 25.0 [16.0–38.0]; ASAT: 26.0 [21.0–37.0]; PAL: 96.0 [73.0–126.0]; ⋎GT: 64.0 [29.0–107.0]
	Creatinin: 70.0 [61.0–83.0]
	CRP: 19.0 [8.5–79.0]
**Microbiology**	Lung: positive in 10/34 (29.4%) patients
	Lymph nodes: positive in 11/21 (52.4%) patients
	Bone: positive in 11/14 (78.6%) patients
	Other: positive in 6/9 (66.7%) patients
**Imaging data (other than PET)**	Thoracic radiography: abnormal in 9/31 (29.0%) patients
	Thoracic CT: abnormal in 28/33 (84.8%) patients
	Abdominal CT: abnormal in 13/27 (48.1%) patients
	Bone MRI: abnormal in 19/19 (100%) patients
**Antibiotherapy**	Isoniazide: 100% of patients
	Rifampicine: 97.6% of patients
	Ethambutol: 95.2% of patients
	Pyrazinamide: 95.2% of patients
	Moxifloxacin: 2.4% of patients
**Treatment duration (months)**	9.0 [9.0–12.0]
**Treatment observance** **>** **80%**	34/36 (94.4%) patients

*Results are expressed as median [IQR] or n (%)*.

#### Healing or Residual Disease Status at the End of Prescribed Therapy

Three of 23 patients (13.0%) had recurrence during the 12-months follow-up after the end of treatment, related to bacterial resistance. According to the criteria indicated in the methods section, 17 patients were cured (including six patients who underwent PET3) and six uncured at the end of treatment (none of them underwent PET3). Lesion by lesion analysis in healed and uncured patients revealed that 16 of 18 initial lymph nodes lesions (88.8%), 10 of 12 initial bone lesions (83.3%), 5 of 6 initial lung lesions (83.3%) were cured at the end of treatment.

### [^18^F]FDG-PET/CT

#### Overall Study Population

PET1 performed at initial diagnosis was completed in 42 Patients. PET2 performed at the end of therapy was completed in 35 Patients. PET3 performed 6 months after the end of therapy was completed in eight Patients. The results of PET1, PET2 and PET3 in all included patients are recorded in Table 2. PET1 was positive in 41 of 42 (97.6%) patients (ΣSUV: 32 [21.1–47.8], MSUV: 6.6 [4.9–9.2]). As compared to data obtained on chest and abdominal CT and MRI (such analysis was possible in 36 of 42 patients), PET1 retrieved unknown additional injured site in 19/36 (52.7%) patients, which were cutaneous lesions in 4/19 (21.0%), liver lesions in 4/19 (21.0%), spleen lesion in 1/19 (5.2%), lung lesions in 2/19 (10.5%), mediastinal lymph nodes in 2/19 (10.5%), and abdominal lymph nodes in 6/19 (31.6%) of cases. Also, among 14 patients with known bone injury, additional bone lesions were discovered on PET1 in three of them (21.4%). Such findings induced a modification of therapy duration in 2/42 (4.7%) patients. Type of medications was not modified and additional CT or MRI examinations were not performed in those two patients. PET2 was positive in 29/35 (82.8%) patients (ΣSUV: 6.1 [3.1–12.5], MSUV 3.1 [2.0–5.0]), and retrieved unknown lymph nodes cervical lesions which were not present at initial diagnosis in 2 of them (5.7%). Such findings did not induce modification of patient management. PET3 was positive in 7 of 8 (87.5%) patients (ΣSUV: 4.0 [2.3–8.0]; MSUV: 2.1 [1.1–4.0]. In the patients who underwent both TEP1 and TEP2, ∑SUV and MSUV values on TEP2 were significantly lower than those calculated on TEP1: 5.4 [0.0–11.2] vs. 30.1 [18.9–43.6] and 2.8 [0.0–4.7] vs. 6.4 [4.7–8.9], respectively, *p* < 0.0001 for both criteria. In the patients who underwent both TEP2 and TEP3, ∑SUV and MSUV on PET2 and PET3 were: 8.0 [3.8–10.9] vs. 3.8 [1.2–7.2] (NS) and 2.8 [2.1–3.8] vs. 1.7 [0.6–3.5] (NS), respectively.

**Table 2:** Tuberculosis locations according to [^18^F]FDG-PET/CT data and values of PET/CT quantitative criteria ∑SUV and MSUV at 3 time points.

		**PET1 (*n* = 42)**	**PET2 (*n* = 35)**	**PET3 (*n* = 8)**
**Presence of [** ^**18**^ **F]FDG abnormal Hot spots evocative of TB lesions**		41 (97.6%)	29 (82.8%)	7 (87.5%)
**Lymph nodes**	Cervical	24 (58.5%)	10 (34.5%)	3 (42.9%)
	Mediastinal	27 (65.9%)	10 (34.5%)	3 (42.9%)
	Axillary	8 (19.5%)	4 (13.8%)	0 (0.0%)
	Abdominal, pelvic	16 (39.0%)	4 (13.8%)	0 (0.0%)
	Inguinal	3 (7.3%)	1 (3.4%)	0 (0.0%)
**Bone**	Spine	14 (34.1%)	4 (13.8%)	0 (0.0%)
	Bassin	6 (14.6%)	1 (3.4%)	0 (0.0%)
	Sup	1 (2.4%)	0 (0.0%)	0 (0.0%)
	Inf	3 (7.3%)	2 (6.9%)	0 (0.0%)
	Other	5 (12.2%)	0 (0.0%)	1 (14.3%)
**Lungs**		11 (26.8%)	5 (17.2%)	1 (14.3%)
**Liver**		3 (7.3%)	0 (0.0%)	0 (0.0%)
**Spleen**		3 (7.3%)	0 (0.0%)	0 (0.0%)
**GUT**		3 (7.3%)	1 (3.4%)	0 (0.0%)
**ENT**		2 (4.9%)	0 (0.0%)	0 (0.0%)
**Muscular**		16 (39.0%)	6 (26.7%)	0 (0.0%)
**Skin**		4 (9.8%)	1 (3.4%)	0 (0.0%)
**Abdominal abcess**		1 (2.4%)	0 (0.0%)	1 (14.3%)
**Other**		4 (9.8%)	2 (6.9%)	3 (42.9%)
**∑SUV**	Mean (std)	39.0 (28.7%)	8.9 (8.2%)	4.7 (3.1%)
(Patients with abnormal PET)	Median (IQR)	32.0 [21.1–47.8]	6.1 [3.1–12.5]	4.0 [2.3–8.0]
**MSUV**	Mean (std)	7.0 (2.8)	3.3 (2.1)	2.8 (2.6)
(Patients with abnormal PET)	Median (IQR)	6.6 [4.9–9.2]	3.1 [2.0–5.0]	2.1 [1.1–4.0]
**∑SUV**	Mean (std)	38.1 (29.0)	7.3 (8.1)	4.1 (3.3)
All patients	Median (IQR)	31.5 [19.6–47.8]	5.4 [0–11.2]	3.8 [1.1–7.1]
**MSUV**	Mean (std)	6.8 (2.9)	2.7 (2.3)	2.4 (2.6)
All patients	Median (IQR)	6.5 [4.8–9.2]	2.7 [0.0–4.7]	1.6 [0.6–3.4]

*PET1, [^18^F]FDG-PET/CT performed at initial diagnosis; PET2, [^18^F]FDG-PET/CT performed at the end of treatment; PET3, [^18^F]FDG-PET/CT performed 6 months after the end of treatment*.

#### Patients With Available Healed or Residual Disease Assessment at the End of Initially Prescribed Therapy

**Figure 2:**
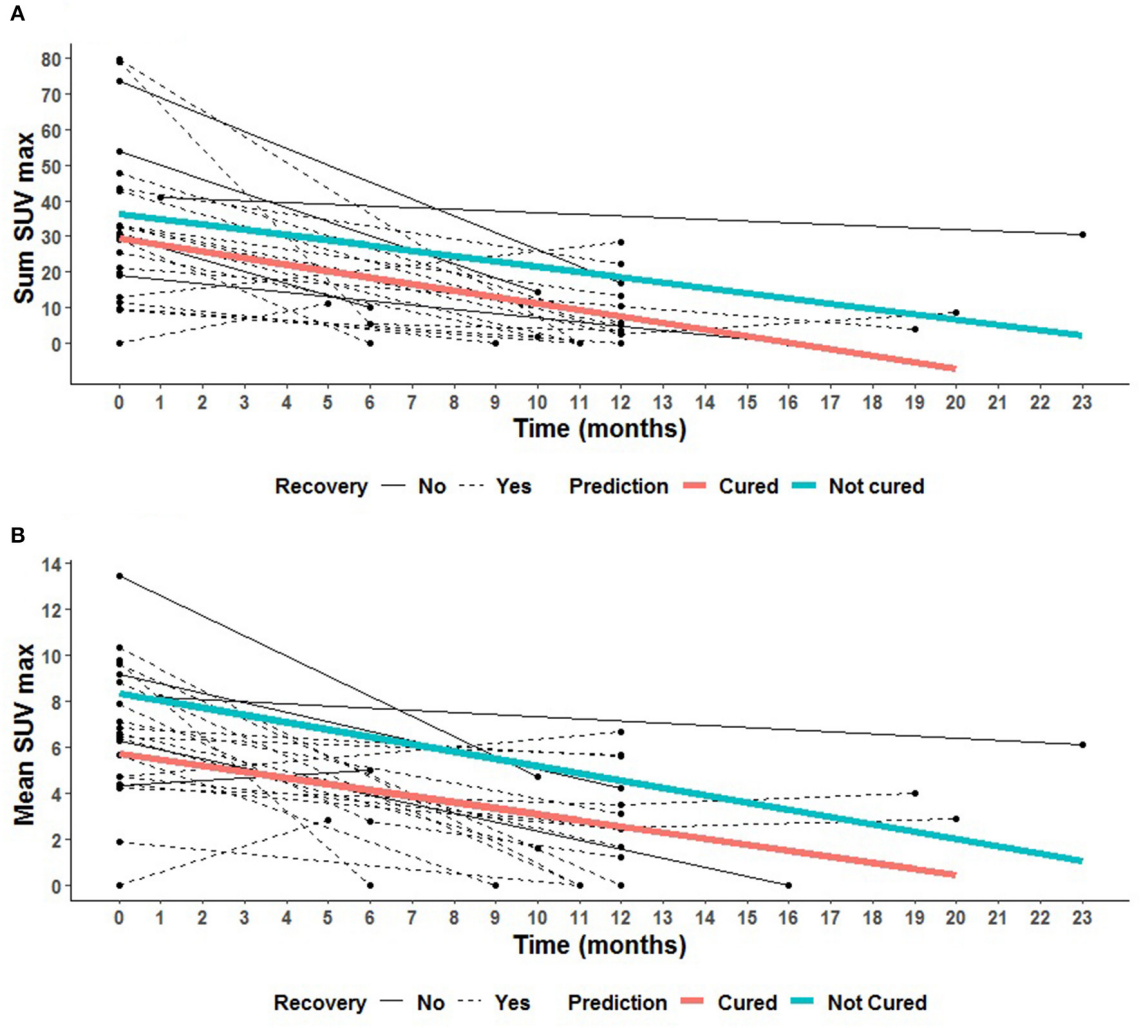
Spaghetti plots of the 23 patients with final diagnosis at the end of treatment. Description of the evolution of the PET parameters with time for each patient, for cured patients (black dotted line) at the end of treatment (red slope, mean prediction), and for uncured patients (black solid line) at the end of treatment (blue slope, mean prediction). **(A)** Description of the evolution of ΣSUV for each patient. **(B)** Description of the evolution of MSUV for each patient.

**Figure 3:**
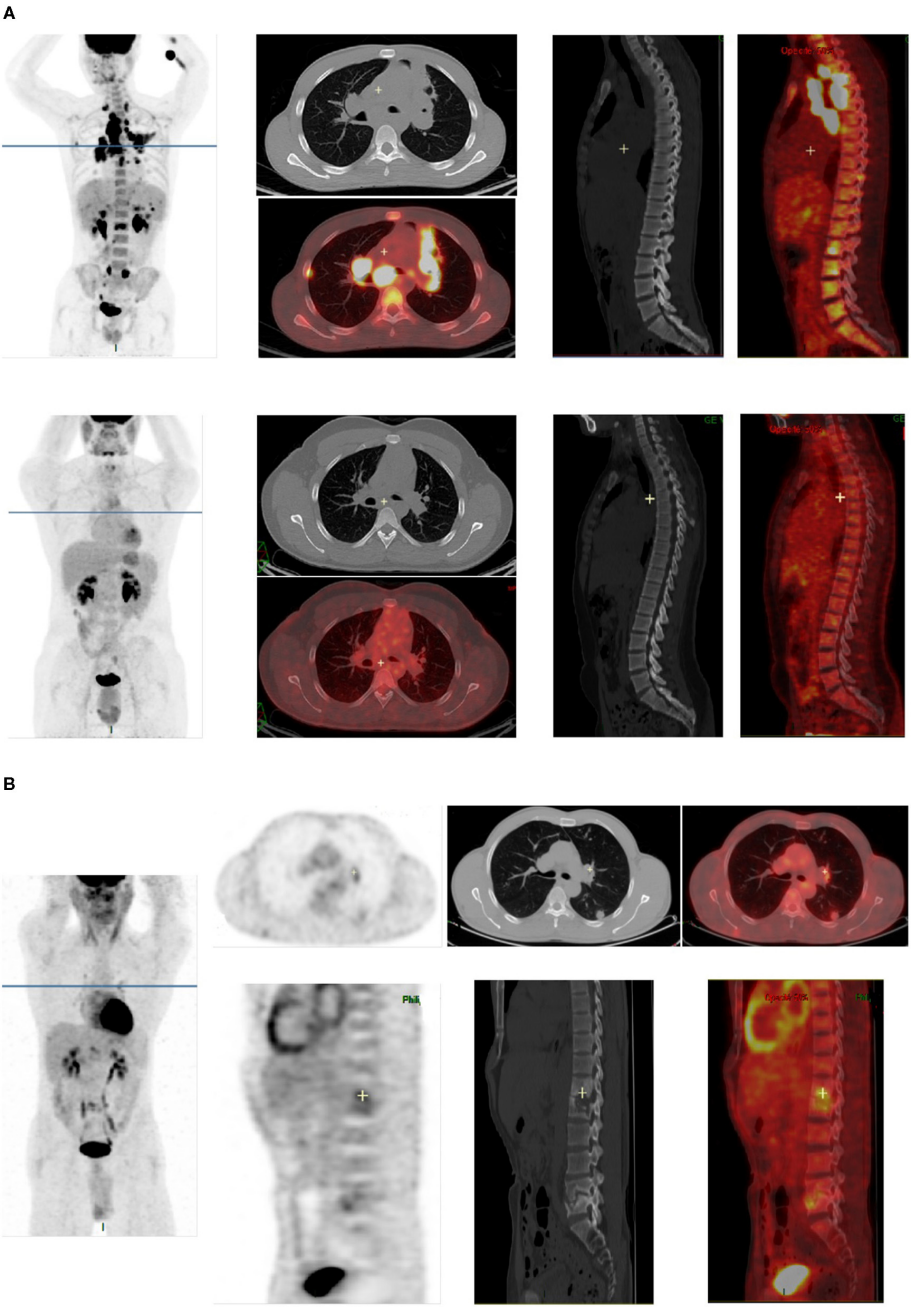
Examples of [^18^F]FDG-PET/CT images in two patients cured at the end of treatment. **(A)** PET1 and PET2 images of a patient cured at the end of treatment. All abnormalities seen on PET1 (lung lesions, mediastinal lymph nodes, bone lesions in dorsal and lumbar spine) completely disappeared on PET2. **(B)** PET2 images of a patient cured at the end of antibiotherapy, showing residual FDG uptake in cured lesions [left lung, left hilar lymph node, and spine (T12, L1, and L5)].

The results of PET1 and PET2 in cured patients (*n* = 17) and uncured (*n* = 6) patients at the end of therapy are recorded in Figures 2, 3. PET1 ∑SUV and MSUV were higher in uncured than in cured patients but the difference was not statistically significant (*p* = 0.55 and 0.19, respectively). Five of six uncured patients had abnormal PET2. Fourteen of 17 cured patients (82.3%) showed persistent [^18^F]FDG uptake in at least one lesion on PET2 (Figure 3). In cured bone lesions, persistent [^18^F]FDG uptake on PET2 was observed in the presence of bone lysis on CT, but not when bone CT was normal. Δ∑SUV and ΔMSUV between PET1 and PET2 were higher but not significantly different between cured and uncured patients: −92.9 [−100.0; −72.3] vs. −73.7 [−77.1; −66.8] (*p* = 0.26), and −70.0 [−100.0; −30.8] vs. −54.3 [−64.9; −25.5] (*p* = 0.47). Lymph node lesions demonstrated a decrease in MSUV (−87.5% [−100.0%; −32.4%]) in cured patient, and conversely an increase in MSUV in uncured patients (+16.3% [−59.7; +37.1]) between TEP1 and TEP2 (*p* = 0.04). Such analysis could not be done with bone lesions since all but 1 bone lesions were cured at the time of TEP2. The variation slope of both criteria (Δ∑SUV and ΔMSUV per month) was not different in cured and uncured patients (Figure 2). On PET3 performed in six cured patients Δ∑SUV and ΔMSUV decreased as compared to PET2, but the differences were not significant.

**Figure 4:**
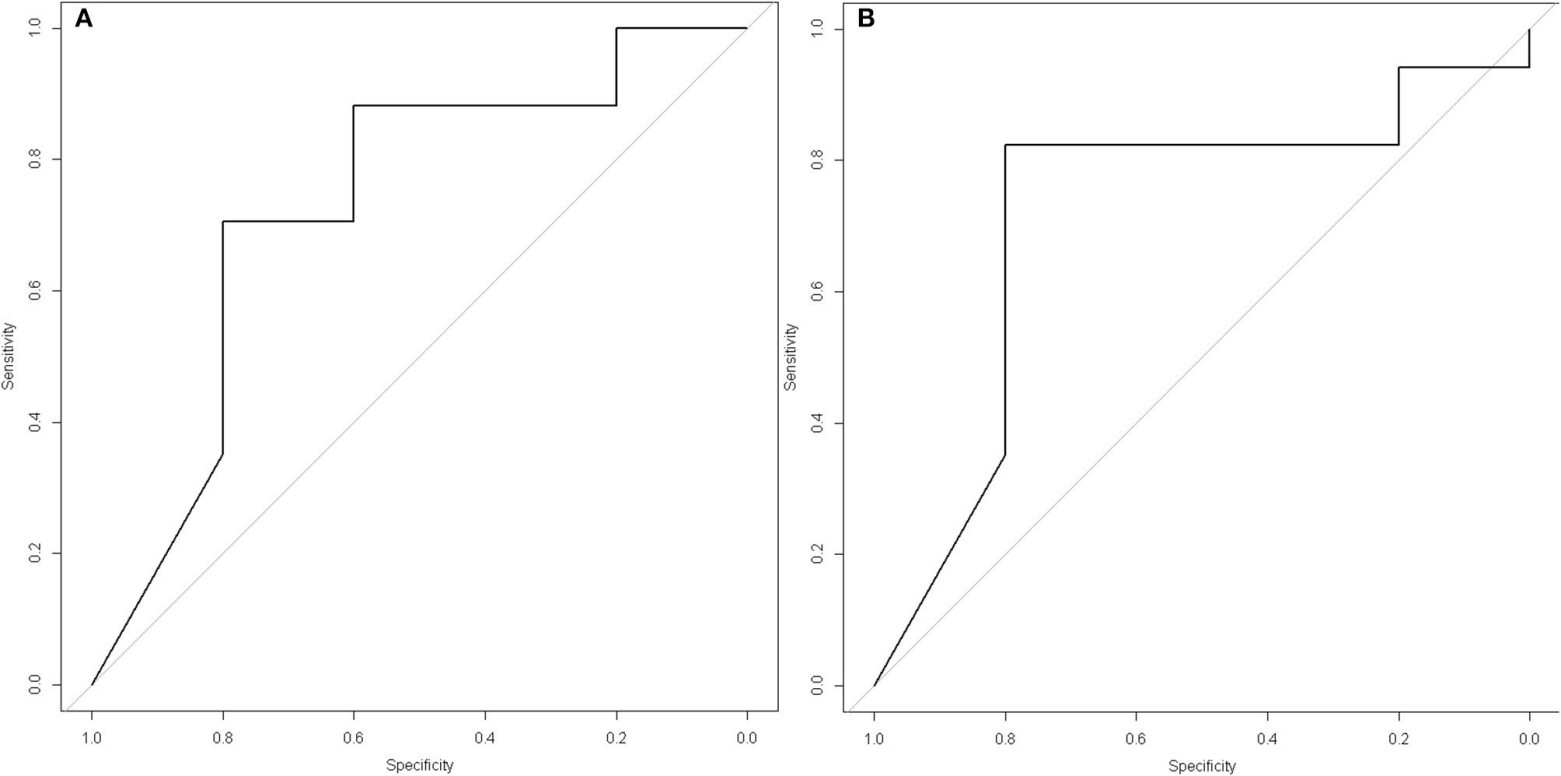
ROC curves with best PET/CT criteria for the diagnosis of healing or residual disease at the end of treatment. **(A)** ΣSUV on PET2 (at the end of prescribed treatment): AUC = 0.73 [0.42–1.00]), Youden index: 5.6, Sensitivity = 64.7 [42.0–87.4], Specificity = 80.0 [44.9–100.0], Positive Predictive Value of ΣSUV <5.6 for the diagnosis of healing = 91.7 [76.0–100.0], Negative Predictive Value of ΣSUV ≥ 5.6 for the diagnosis of healing= 40.0 [9.6–70.4]. **(B)** MSUV on PET2: AUC = 0.72 [0.42–1.00]), Youden index: 3.5, Sensitivity = 76.5 [56.3–96.6], Specificity = 80.0 [44.9–100.0], Positive Predictive Value of MSUV <3.5 for the diagnosis of healing = 92.9 [79.4–100.0], Negative Predictive Value of MSUV ≥ 3.5 for the diagnosis of healing = 50.0 [15.3–84.6].

ROC curves were performed for all quantitative criteria. ∑SUV and MSUV on PET2 showed the highest AUC on ROC curves for the diagnosis of healing or residual disease at the end of treatment (0.73 [0.42–1.00] and 0.72 [0.42–1.00], respectively) (Figure 4). MSUV under 3.5 had a sensitivity of 76.5% [56.3–96.6%], and a specificity of 80.0% [44.9%; 100.0%] to affirm healing at the end of treatment. The probability to be healed at the end of treatment for a patient with MSUV <3.5 on PET2 was 92.9%. The probability to have residual disease at the end of treatment for a patient with MSUV ≥ 3.5 on PET2 was 50%.

The lesion by lesion analysis of PET2 in healed and uncured patients at the end treatment showed that when the highest SUV_max_ value was considered with a threshold of 3.5, healing could be correctly affirmed (SUV_max_ <3.5) on PET2 for 11/16 (68.7%) patients, 13/16 (81.2%) lymph node lesions, 8/10 (80.0%) bone lesions, 5/5 (100%) lung lesions, and 3/5 (60%) muscular lesions; and residual disease could be correctly detected (SUV_max_ ≥ 3.5) in 4/5 (80%) uncured patients. In uncured patients, all initial lymph nodes (*n* = 4) and bone (*n* = 2) lesions showed SUV_max_ > 3.5 on PET2, whereas all initial muscular lesions (*n* = 5) showed SUVmax <3.5 on PET2. This suggests that residual disease was still present at the end of treatment in all initial lymph nodes and bone lesions, whereas all muscular lesions had cured. The probability for a patient to be healed when SUV_max_ was <3.5 on PET2 was 11/13 (84.6%). The probability for a patient to be uncured when SUV_max_ was ≥ 3.5 on PET2 was 4/10 (40%).

## Discussion

[^18^F]FDG-PET/CT at diagnosis was positive in 97.6% of the patients and discovered unknown lesions in 52.7% of cases. ∑SUV and MSUV clearly decreased on PET2 at the end of treatment in cured patients, but abnormal hot spots persisted in 82.3% of them. ∑SUV and MSUV did not decrease between PET2 and PET3 in cured patients. MSUV under 3.5 on PET2 was the best criteria to diagnose healing at the end of treatment.

The sensitivity of [^18^F]FDG-PET/CT at initial diagnosis in our study is comparable to those previously reported (97–100%) (8, 9, 12–14). Additional unknown injured sites were discovered on PET1 in 52.7% of cases. Such parameter is highly variable in the literature, from 10 to 69%, probably depending on the study population (disseminated or local tuberculosis). Discovered additional sites mainly concerned cutaneous lesions, liver lesions and abdominal lymph nodes not detected on CT. As compared to data of the literature (8, 9, 12–14), the percentage of residual [^18^F]FDG uptake in lesions at the end of antibiotherapy is higher in our study (82 vs. about 40–50% in most studies). This may be explained by the characteristics of the study population: only extra-pulmonary and mostly disseminated tuberculosis in our study when other studies included both pulmonary and other types of tuberculosis. The cured lesions which remained positive on PET2 were lymph nodes, bone, lung, muscle lesions or subcutaneous abcesses. In cured bone lesions residual [^18^F]FDG uptake was observed in the presence of bone lysis on CT but not when bone CT was normal. This is concordant with residual [^18^F]FDG uptake being related to bone repair after sterilization. The data observed in lung lesions are surprising and emphasize the role of BK tubage to monitor therapy in pulmonary locations.

Despite the small number of patients analyzed during post-treatment follow-up (*n* = 8, 6 cured at the end of therapy), we could conclude that [^18^F]FDG-PET/CT does not normalize 6 months after the end of antibiotic therapy: in seven patients TEP3 remained positive, with comparable MSUV as compared to PET2. Many reasons may explain such data: long healing process with [^18^F]FDG uptake by activated fibroblasts, latent tuberculosis with the persistence of live but non replicating bacteria, the persistence of dead bacteria or of products of bacterial lysis inducing persistent immune-reactive inflammation. It is probable that a 6 months delay is too short and that a more delayed [^18^F]FDG-PET/CT scan would have returned negative. This is supported from previous data of our department in six patients cured since 3 years who had all a negative [^18^F]FDG-PET/CT scans. However, a longer delay than 6 months after treatment to confirm healing is not applicable in the clinical situation since most of the patients are difficult to follow. With this regards, it is worth noting that only 23 of 42 patients completed the entire study course, many of being absent to 1 or 2 of the 3 [^18^F]FDG-PET/CT appointments.

Six of the 23 patients who completed entire follow-up were not cured at the end of initial antibiotic therapy. Non observance of treatment can be incriminated in 1 of those. This confirms a posteriori the need for a marker of healing at the end of treatment. Also, lesion by lesion analysis in healed and uncured patients revealed that 16 of 18 initial lymph nodes lesions (88.8%), 10 of 12 initial bone lesions (83.3%), and 5 of 6 initial lung lesions (83.3%) were cured at the end of treatment. This suggests that the location (lymph nodes, bone, or lung) of initial lesions is not related to the persistence of residual disease at the end of treatment.

Despite this small number of uncured patient we could identify SUV_max_ ≥ 3.5 on PET2 as a criteria to identify uncured patients with good sensitivity (76.5%) and specificity (80.0%). SUV_max_ on PET2 was also previously reported as a valuable criteria for healing assessment by Sathekge et al. (15). However these authors found a threshold value of 4.5 for SUV_max_. This emphasizes the need of other studies to confirm the value of this criteria, and refine the value of the threshold to be considered. ∑SUV on PET2 also showed a high AUC on ROC curves for the diagnosis of healing or residual disease at the end of treatment (0.73 [0.42–1.00]) (Figure 4). This suggests that the extension of initial disease plays a role in healing or not, the patients with extensive disease being more at risk for residual disease at the end of treatment. Other criteria which had to be further studied are the ∑SUV per month and the ΔMSUV per month which theoretically take into account the problems of non-observance, but they were not different in cured and uncured patients (Figure 2). It is worth noting that the variation of ∑SUV and MSUV between initial diagnosis and end of antibiotic therapy did not appear as reliable criteria according to ROC curves. This may be related to statistical reason related to the small number of patients.

Also we found that ∑SUV has no additional value as compared to MSUV, despite it takes into account the extension of the disease. This is probably due to the facts that only one active lesion is enough for the patient to be uncured, and that there is no relation between the number of lesions at initial diagnosis and the number of uncured lesions at the end of therapy.

We did not use other PET criteria than SUV_max_ (sum and mean) because other PET criteria such as MTV and TLG need to determine the volume of each lesion: this was not feasible for technical reasons in most patients who demonstrated multiple (especially lymph node) lesions. Also SUVpeak was not available in all analysis software at the time of the study so we could not use it.

Overall this study, like others in this field, support present interest for new radiotracers more specific for infection, especially those specifically targeting live bacteria (8, 16). Unfortunately until now no specific marker of mycobacterium has been radiolabeled for *in-vivo* imaging. Authors suggest that [^18^F]fluoro-choline may be of interest in this setting (12). It showed lower uptake in BK lesions than [^18^F]FDG but was never evaluated in therapeutic response assessment. Maybe the number of falsely positive sites at the end of treatment would be lower with [^18^F]fluoro-choline than with [^18^F]FDG.

### Limitations of the Study

This study is hampered by the small effective of patients who completed the entire protocol, affecting the strength of statistical tests. This is probably due to the social characteristics of most patients whose follow-up was difficult (foreign origin with language barrier, homeless patients).

Additionally the study was performed at five different nuclear medicine centers, using three different PET/CT tomographs. Unless each PET/CT tomograph was calibrated according to manufacturers' specifications, harmonization of the data through a phantom study was not performed. Therefore, despite being very promising, the cut-off values identified on PET2 may be currently only preliminary data, which need further validation in the future.

Finally, the evaluation of other PET quantitative criteria, such as MTV and TLG could not be performed at the time of the study, which could be of interest in this setting.

## Conclusion

[^18^F]FDG-PET/CT at diagnosis was positive in 97.6% of patients with confirmed lymph node or bone tuberculosis, and discovered unknown lesions in 53.7% of cases. ∑SUV and MSUV clearly decreased on PET2 at the end of treatment in cured patients, but abnormal hot spots persisted in 82.3% of cases. SUV_max_ on PET2 was the best criteria to discriminate between healing and residual disease at the end of treatment, with a threshold of 3.5 in our study which needs further validation in the future.

## Data Availability Statement

The original contributions presented in the study are included in the article/supplementary files, further inquiries can be directed to the corresponding author/s.

## Ethics Statement

The studies involving human participants were reviewed and approved by the French Ethic Committee CCP Ile-de-France 1 Hôtel Dieu, 1 place du parvis Notre Dame, 75004 Paris (ID-RCB: 2011-A01658-33). The patients/participants provided their written informed consent to participate in this study.

## Author Contributions

LS-M, JK, CR, PY, and CLa designed the study and obtained funding. AL, FMé, SN, OD, TG, and DP included and followed the patients. LS-M, KB, NM, MS, and CLe performed centralized interpretation and scoring of PET/CT scanners. TA and CLa performed statistical analyses. LS-M, TA, and CLa wrote the manuscript. LS-M, TA, CLa, CR, FMo, and ED reviewed and validated the manuscript. All authors contributed to the article and approved the submitted version.

## Conflict of Interest

The authors declare that the research was conducted in the absence of any commercial or financial relationships that could be construed as a potential conflict of interest.

## Publisher's Note

All claims expressed in this article are solely those of the authors and do not necessarily represent those of their affiliated organizations, or those of the publisher, the editors and the reviewers. Any product that may be evaluated in this article, or claim that may be made by its manufacturer, is not guaranteed or endorsed by the publisher.
